# Assessing the value of eHealth for bariatric surgery (BePatient trial): study protocol for a randomized controlled trial

**DOI:** 10.1186/s13063-018-3020-x

**Published:** 2018-11-14

**Authors:** Dirk P. A. Versteegden, Magaly J. J. Van himbeeck, Simon W. Nienhuijs

**Affiliations:** Department of Surgery, Catharina Hospital, Michelangelolaan 2, 5623 EJ Eindhoven, the Netherlands

**Keywords:** Telehealth, eHealth, Bariatric surgery, Gastric bypass, Gastric sleeve, Telemedicine technology

## Abstract

**Background:**

The expansion of digital devices and widespread access to the Internet has opened up opportunities to provide patients with more personal information. It can be hypothesized that eHealth in addition to standard care could enhance clinical outcomes such as increased weight loss, co-morbidity reduction, and commitment to the program. The beneficial value of incorporating eHealth applications as standard postoperative care is yet to be established. In this trial, the value of different levels of eHealth are assessed.

**Methods/design:**

Two hundred adult patients with a body mass index (BMI) ≥ 40 kg/m^2^, or ≥ 35 kg/m^2^ with obesity-related co-morbidity, undergoing sleeve gastrectomy or gastric bypass will be enrolled in this randomized controlled trial. Patients will be randomly assigned to one of the groups: receiving standard care (control group, *n* = 100); have access to an online eHealth platform in addition to the previous group (online group, *n* = 50); or receive wireless monitoring devices in addition to previous groups (device group, *n* = 50). The total follow-up period is two years postoperatively. Primary outcome is weight loss in terms of BMI. Secondary outcomes include: quality of life; return-to-work time; co-morbidity reduction; additional contacts; and ease of use of devices.

**Discussion:**

In this trial, the value of different levels of eHealth will be assessed. This addresses an important aspect of a changing healthcare environment.

**Trial registration:**

Trialregister.nl, NTR6827. Retrospectively registered on 19 November 2017. http://www.trialregister.nl/trialreg/admin/rctview.asp?TC=6827.

**Electronic supplementary material:**

The online version of this article (10.1186/s13063-018-3020-x) contains supplementary material, which is available to authorized users.

## Background

Bariatric surgery is the only treatment with long-standing effect on morbid obesity. The key elements to success are the patient selection, an experienced bariatric team, and a completed follow-up program. Follow-up programs can consist of, for example, providing social support in support groups, teaching psychological skills, such as coping with the body change, or teaching self-regulation of body weight [1–5]. Furthermore, follow-up is important for dietary and sports counseling [2, 5]. The experience of the team members and coaching skills are essential in indicating the suitable procedure if necessary and guiding patients through the process. Various studies have found a significant positive effect of a completed follow-up program after bariatric surgery on maintaining weight loss [6, 7]. There is a burden for this on-site provided care as organizational and financial resources are not unlimited, especially as the follow-up period is an obligatory five years or, if possible, lifelong. Even if this aftercare is provided, not all patients complete the entire program. Various reasons are possible for an increasing no-show rate; loss of enthusiasm for on-site visits could be one of them.

Analogous to other chronic diseases, the addition of telehealth could be useful for the treatment of obesity. Telehealth is the delivery of health-related services and information via telecommunication technologies. It encompasses preventative, promotive, and curative aspects. Examples are exchanging health services or education via videoconference, transmission of medical data for disease management (remote monitoring) and advice on prevention of diseases, and promotion of good health by patient monitoring and follow-up. Participation with eHealth has been investigated and considered useful in the treatment of obesity [4, 8–13]. In a systematic review, self-measured blood pressure monitoring was associated with better control of hypertension in the first year [14]. Its value in a bariatric trajectory has not been investigated. It can be hypothesized that self-control by eHealth could enhance clinical outcome through more weight loss and co-morbidity reduction. Long-term realistic goal setting, consistent use of routines, and self-monitoring have been proven effective for weight loss maintenance [15]. Patients with higher self-control are more confident regarding their abilities, which leads to higher commitment and adherence to the program. This eventually leads to more weight loss  [4]. For this purpose, an online monitoring program was designed for our obesity department to provide preoperative information as well as aid in the post-bariatric phase by self-control wireless devices for registration of biometric outcomes, teleconference opportunities, and access to additional information.

The objective of this trial is to determine whether patients benefit from different degrees of eHealth and self-monitoring. This research will contribute to the still small body of evidence around the value of eHealth.

## Methods/design

### Study aim

The aims of the BePatient trial are to assess the value of eHealth by comparing different levels of telehealth provided to bariatric patients. It is hypothesized that the more eHealth is provided, the higher postoperative weight loss will be, the less likely additional contacts/visits will be consumed, the higher the patients’ satisfaction will be, and the more benefits in co-morbidity will be found. The addition of eHealth provides the patients more self-control, which could result in better commitment to the follow-up program. The primary study hypothesis is that the benefit of adding eHealth to a bariatric care program would lead to a larger reduction in body mass index (BMI) two years after the procedure compared to standard care.

Two main research questions are addressed:

1. Does an eHealth platform increase weight loss after bariatric surgery?
2. Does providing patients with electronical wireless monitoring devices lead to more weight loss?

### Study setting

This open-label randomized controlled trial is conducted at the Catharina Hospital Eindhoven, a large teaching hospital in the Netherlands. Around 1000 bariatric procedures are performed annually by dedicated bariatric surgeons.

### Study registration

The study was reviewed and approved by the institutional medical ethical board. The protocol (protocol identification number: NL56992.100.16, version 3, 11 July 2016) conforms to the principles of Good Clinical Practice and the Declaration of Helsinki. Table 1 shows a structured summary conforming with a standard WHO trial registration dataset. Retrospective registration was achieved in the Dutch trial registration on 19 November 2017 (Identifier number NTR6827.) The trial is ongoing and currently recruiting. The first patient was included on 21 February 2017. In case of amendments to the protocol, the institutional medical ethical board will be notified for approval. This protocol was conducted conforming the SPIRIT 2013 Checklist (Additional file 1).

**Table 1:** WHO trial registration dataset – structured summary

Data category	Information
Primary registry and trial identifying number	Nederlands trial register - NTR6827
Date of registration in primary registry	19 November 2017
Secondary identifying numbers	Clinicaltrials.gov: NCT03394638 Protocol number: NL56992.100.16
Source(s) of monetary or material support	N/A
Sponsor	N/A
Contact for public/scientific queries	Obesity Center, Catharina Hospital Eindhoven, The Netherlands
Public title	BePatient trial
Scientific title	Assessing the value of eHealth for bariatric surgery (BePatient trial): study protocol for a randomized controlled trial
Country of recruitment	The Netherlands
Health condition(s) or problem(s) studied	Morbid obesity
Intervention(s)	Standard care vs eHealth platform vs eHealth platform + self-monitoring devices
Key inclusion and exclusion criteria	Age > 18 years; eligible for bariatric surgery; BMI > 40 kg/m^2^ or > 35 kg/m^2^ with related co-morbidity; ongoing access to the Internet; ability to use smartphone or tablet; ability to understand Dutch language; signed informed consent
Study type	Open randomized controlled trial
Date of first enrollment	21 February 2017
Sample size	200
Recruitment status	Recruiting
Primary outcome(s)	BMI
Key secondary outcomes	Sociodemographics, weight, co-morbidity status, quality of life, return to work, satisfaction and commitment, inventory of use of additional support, devices and data-traffic
Ethics review	Reviewed and approved by Institutional Review Board on 15 August 2016
Summary results	Planned to be released in 2020
IPD sharing statement	Undecided

### Study population

**Fig. 1:**
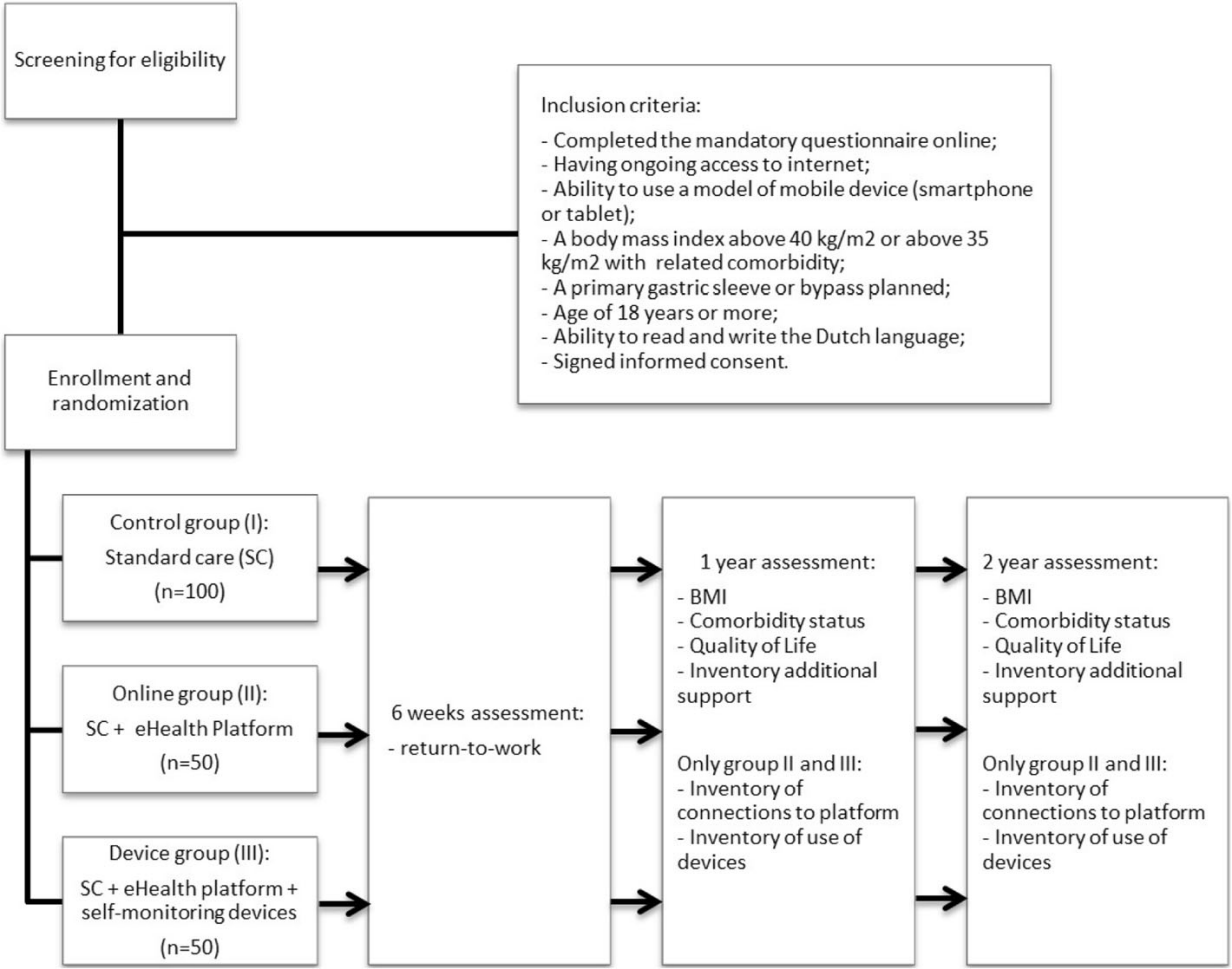
*Flowchart* illustrating the recruitment and allocation process in this trial

**Fig. 2:**
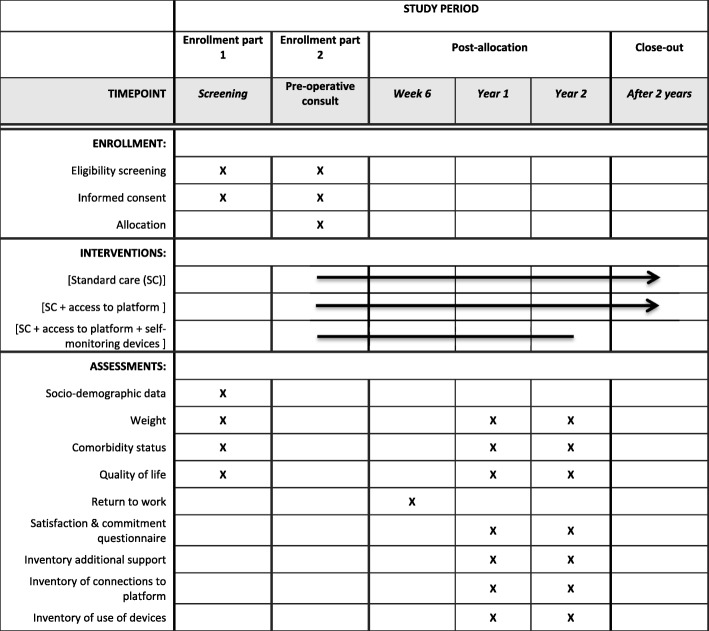
Standard Protocol Items: Recommendations for interventional Trials (SPIRIT) figure

Obese adults with a BMI > 40 kg/m^2^, or > 35 kg/m^2^ with at least one obesity-related co-morbidity, such as hypertension or diabetes, unsuccessful previous attempts to lose weight, and willingness to attend a follow-up program could be indicated for a bariatric procedure. Patients referred to the Catharina Obesity Center are asked for access to the Internet and willingness to complete the mandatory screening questionnaire online. From the beginning of 2015, this was the standard procedure of the center. In case of inability or unwillingness to use the online version, the patient receives a postal alternative. After returning the questionnaires, patients are invited to the outpatient department. There they receive a presentation and interview by an obesity nurse, dietician, psychologist, and physiotherapist and receive extensive blood tests. These results are discussed in an obesity team with bariatric surgeons. After approval for the operation by this multidisciplinary team, patients visit the bariatric surgeon. If the result of this consult is a planned operation, the patient could be considered eligible for participation to this study. The sleeve gastrectomy and bypass are by far the most performed initial procedures at this center. Therefore, only patients with approval for a primary gastric sleeve or bypass are finally considered to be eligible. Figure 1 shows a flowchart illustrating the recruitment and allocation process in this trial. Figure 2 shows the SPIRIT schedule of enrollment, interventions, and assessments used in this trial.

### Inclusion criteria

The inclusion criteria to participate in this study are:

- Completed the mandatory questionnaire online;
- Having ongoing access to the Internet;
- Ability to use a model of mobile device (smartphone or tablet);
- A BMI > 40 kg/m^2^ or > 35 kg/m^2^ with related co-morbidity (hypertension, diabetes type 2, hyperlipidemia, obstructive sleep apnea syndrome, or arthralgia of lower limbs;
- A primary gastric sleeve or bypass planned;
- Age ≥ 18 years;
- Ability to read and write the Dutch language; and
- Signed informed consent.

Those who did not met the abovementioned inclusion criteria were excluded.

### Inclusion procedure

Patients are informed about the study at two separate times. During the screening phase, patients receive a leaflet with information about the study and are informed during a group meeting with other patients. Later, if a procedure is planned and the operation date is known, patients are again informed by a trained member of the research team in an individual consultation. Afterwards patients are given the time to consider participating. If they decide to participate, written informed consent is obtained (see Additional file 2). Patients are informed that they can stop at any time without any effect on their usual care and without being required to provide a reason for stopping. After completion of the study, patients will receive standard care. There is no financial benefit or other compensation for participating in this trial.

### Randomization and treatment allocation

Participants who fulfill all selection criteria and signed the informed consent form are randomized in a 2:1:1 ratio. Simple randomization is used, meaning every individual has an equal chance to be enrolled in one of the three groups. Allocation is into one of three groups: (1) control group (*n* = 100); (2) platform group (*n* = 50); and (3) device group (*n* = 50). This randomization is computer-generated in a Care Coordination Module functionality in the BePatient platform. Allocation concealment will be ensured, as the allocation of patients is generated after the patient signs the informed consent form and thereby included in the study.

### Safety assessment

Severe adverse events (SAE) within this study are assumed to be difficult to determine ahead of time. Personal injury through use of the devices is the only predetermined category; other AEs will be identified upon the decision of the treating team. In case of an AE, this will be reported to the ethics committee and included in the results. Due to the expected absence of any SAE, there is no annual safety report or monitoring board.

### Interventions

#### Standard care

All patients will receive standard care which consists of a five-year follow-up scheme after the bariatric procedure. The first year consists of around 10 individual and three group consultations. These follow-up visits are usually at around 1, 3, 6, 8, 9, 10, 12, 16, 20, and 24 months postoperatively and include consultations with their surgeon, dieticians, physiotherapists, and obesity nurses. After the first year, visits become less frequent. If deemed necessary, additional contact can be made. During the total follow-up time, blood markers are frequently assessed.

#### eHealth platform

In addition to the standard care, patients in the online and device groups will have continued access to the patient platform. The Catharina BePatient website is a bariatric online module developed by BePatient™, personalized to the hospital’s standard. This company designs eHealth solutions and invents new patient pathways. In 2014 they developed an online program for bariatric patients. This encompassed shared personal health records with remote patient monitoring through wireless devices, assessment of indicators on the patients’ real life, providing a social network and coaching between healthcare professionals and patients and e-learning of selected information to raise awareness of therapeutic education. The module was complete in June 2014. Thereafter, specific questionnaires were added and personalized with color and design standards of the Catharina Hospital, resulting in the Catharina BePatient website. This website has two types of user accounts: one for the patient and one for the healthcare provider. For the latter, it can be used to modify website content and download patient data and data-traffic information. Since the start of 2015, all new patients receive registration details to create a personal account. After patients register, they are asked to sign the user agreement, which includes gathering of data and providing access to these data by the medical team. Hereafter, patients can fill in the mandatory questionnaires which are used for screening purposes, as described before. Upon completion of the questionnaires, patients are able to access additional content on the platform, depending on their stages in the treatment. A variety of content can be found on the platform, including: dietary tips; how to use medication; frequently asked questions; obesity-related facts; instruction videos of several physical exercises; and more. Users can also make a list of their eating habits which they can bring to the next appointment. Regularly, the information is revised by healthcare professionals of the Catharina Hospital to make sure information is up-to-date. Furthermore, new information and lectures are added frequently.

#### Self-monitoring devices

In addition to standard care and access to the eHealth platform, patients in the device group also have access to four wireless telemonitoring devices: a weighing scale; a blood pressure meter; oxygen saturation meter; and an activity bracelet. All devices are connectable with most smartphones and tablets using Bluetooth. By doing this, they can view their measurements on dedicated mobile applications. This can be displayed as a list of measurements or in a graph. By doing this they can track their progress in, for instance, weight loss. Patients can also set goals to achieve, for instance, a total number of steps or distances walked. These measurements are also visible on their platform accounts once they synchronize the devices to their BePatient accounts. This gives the research team insight in the frequency of use of the devices.

## Assessment of study outcomes

### Primary outcome measure

#### BMI

The primary outcome measure will be BMI. BMI is calculated with the formula: weight in kg / (height in m)^2^ = kg/m^2^. BMI is recorded through the whole follow-up scheme of five years which is standard care in this hospital. The weight of participants will be measured on calibrated electronic clinical weighing scales while fully clothed (without jackets or other items which can be removed quickly). For this study, BMI will be reported at one and two years postoperatively.

### Secondary outcome measures

#### Co-morbidity status

Co-morbidity status of diabetes mellitus type 2, hypertension, dyslipidemia, obstructive sleep apnea syndrome, gastro-esophageal reflux disease, and arthralgia is assessed at baseline and at one and two years postoperatively. A co-morbidity is considered in remission if patients do not experience any complaints and if no medication was needed since their last visit. When patients experience fewer complaints or require less medication, the co-morbidity is considered improved. If no changes are seen or when the co-morbidity worsens, it is regarded as unimproved.

#### Quality of life

Health-related quality of life is measured at baseline and at one and two years postoperatively using a Dutch translation of the RAND36 questionnaire [16]. The RAND36 consists of 36 questions derived into nine domains: physical functioning; social functioning; physical role limitations; emotional role limitations; mental health; vitality; pain; general health perception; and health change perception. For each domain, a score of 0–100 is calculated. A low score corresponds with poor health-related quality of life.

#### Return to work

The time it takes for patients to recover until they are fit to work is assessed using a questionnaire six weeks postoperatively.

#### Inventory of additional support

It can be expected that some patients who are randomized in the control or online group will make use of additional support or choose to purchase or use self-monitoring devices themselves. Patients are not limited to seek additional support if they deem that necessary (i.e. mental coaching, sporting activities, Internet forums/groups, other patient platforms, and other self-monitoring devices). To gain insights into this, a yearly questionnaire will be used, which includes questions about the frequency of the use of the abovementioned additional support.

#### Inventory of connections to platform

Information about the data traffic is gathered and stored on the platform. The number of connections is recorded as well as number of page views, time spent per session, and time of the day of the connection. Furthermore, the medium used to access the content (i.e. if the content is accessed on a web browser, mobile phone, or mobile app) is also recorded.

#### Inventory of use of devices

The number and frequency of measurements for each device will be recorded.

#### Satisfaction and commitment questionnaire

Measuring program commitment is assessed yearly with a six-item questionnaire adapted from a version used by Neubert and Cady in 2001 [17]. This includes six yes-or-no questions about their satisfaction and commitment towards the treatment program.

### Data collection

Sociodemographic and clinical information is gathered in the standard follow-up program noted in the patients’ electronic hospital files and is accessible to the treatment team. This includes: sex; age; weight; BMI; status of co-morbidities; quality of life; and blood markers. Additional parameters, such as results of questionnaires, are not normally used in the follow-up program and are therefore stored at a secured part in the Care Coordination Module functionality on the BePatient platform. To analyze the data, individuals are coded in order of registration, starting with 00–001. Members of the research team are responsible for the maintenance and monitoring of completeness and correctness of the data. An annual status report will be sent to the institutional ethical commission.

### Statistical analysis

#### Sample size calculation

Lack of standardization for measuring the value of eHealth makes defining relevant endpoints difficult. From a clinical point of view, surgical success is defined as a total weight loss > 20%. The patients not achieving this success could especially benefit from eHealth. Furthermore, there is a group with initial weight loss and subsequent weight regain ending in the same group of failures. In a report from The Longitudinal Assessment of Bariatric Surgery Consortium, which is a multicenter observational cohort study, the results of 2458 patients were reviewed [18]. To evaluate common patterns of weight change from baseline to three years among participants following a gastric bypass, five weight-change trajectory groups were identified. A slight regain of weight occurred in every trajectory group, two years after the procedure different patterns could be distinguished. In another report, trends in weight regain following gastric bypass were reviewed (smaller group, longer follow-up) [19]. Weight gain was found to be a common complication, on average in the range of 21–29% of lost weight. Excessive weight gain was experienced by over one-third of patients. Greater initial absolute weight loss leads to more successful long-term weight outcomes. As the patterns of trajectory groups could be distinguished at two years after the operation with a difference of 10% weight loss, a hypothesis was composed. The difference of 10% corresponds to an arbitrary 4 BMI points. It is, therefore, hypothesized that the addition of eHealth would reduce the BMI by another 4 point at two years postoperatively.

This trial studies the continuous response variable BMI from independent control and experimental participants with two controls per experimental individual. If the true difference in the experimental and control means changes is 4 BMI points with a standard deviation of 8, 48 experimental individuals and 96 controls need to be studied (equal variances assumed) to be able to reject the null hypothesis that the population means of the experimental and control groups are equal with power = 0.8 and α = 0.05. In this trial, two experimental groups are defined. Therefore, at least a total of 200 patients will be enrolled in this trial, assuming no more than 4% missing data.

#### Data analysis

Descriptive statistics of the data will be performed for all baseline characteristics and outcome measures. Groups will be compared using ANOVA-tests and χ^2^-tests (normal distribution) or Mann–Whitney *U*-tests (non-normal distribution) to analyze outcome measures. Comparisons of baseline characteristics will be done descriptively. Due to the repeated measure nature of the design (preoperative, one year postoperatively, and two years postoperatively) repeated measure ANOVA will be used to analyze weight-loss patterns between the intervention groups and over time.

SPSS v.25.0 (IBM Corp., Armonk, NY, USA) is used for statistical analysis and handling of data. Analysis will be performed using the intention-to-treat principle. A *p* value < 0.05 (two-sided) will be considered statistically significant. Subgroup analysis may help identify subgroups in which interventions work better or worse. Therefore, predetermined subgroup analysis will be performed. A subgroup analysis will be done comparing active platform or device users with inactive users to determine if activity influences outcome. Also, several age groups will be analyzed, as older patients might have a harder time working with electronical device and the online platform. Finally, a subgroup analysis will be performed comparing gender.

Substantial amounts of missing data will be handled using multiple imputations if deemed necessary.

Outcomes are planned to be published in a peer-reviewed journal upon completion of this trial.

## Discussion

Over the last decades, a lot has changed in healthcare, from both the perspective of the patient as well as that of the healthcare professional. On the side of the healthcare professional, almost everything has been digitalized which, most of the time, makes working efficiently easier, for instance, viewing patients’ history or blood values. On the other hand, a lot has changed on the side of the patient as well. Nowadays the vast majority of patients in western countries have access to the Internet and mobile phones. This provides patients with a way to quickly find information about their health status, disease, and treatment. However, this information is not always correct and is frequently not applicable to individual patients. This can lead to misinformed patients. Making sure that patients are correctly informed is one of the most important aspects in healthcare.

A dedicated eHealth platform can aid or support communication towards patients. However, the value of eHealth is not yet established. Reviews on the efficacy of eHealth interventions for weight loss and weight loss maintenance show promising effects [20–22]. This trial is the first randomized research that evaluates the effects of different levels of eHealth compared to a control group after bariatric surgery.

Some limitations need to be addressed as well. As mentioned above, it can be expected that some patients will make use of other eHealth solutions or use self-monitoring devices of their own. This is, of course, dependent on the patients’ own desire and cannot be restricted. This can possibly lead to bias. To limit the effects of this bias, patients are asked if they made use of other support or devices other than the ones provided in this trial. On the other hand, some patients who enroll in one of the intervention groups might not make use of said intervention. Disinterest and lack of time can be reasons for this. Outside the context of this study, there will always be differences in the commitment to a follow-up program between patient.

The success of bariatric surgery depends on motivation. An attempt to increase this motivation is to add eHealth applications and self-monitoring devices. However, study patients can experience this as mandatory which can imply a counterproductive effect on their incentive. In this respect, patients are informed very well about the voluntariness to use the devices and patient website. However, patients may lose interest after some time during the total lifespan of the study of two years. Also, another possible drawback of the use of devices can be that they generate an opposing effect on patients. The increased awareness of their weight can lead to some sort of compulsive need to keep losing more weight. We advise patients to not weigh themselves on a daily basis because natural changes in their weight can result in a false understanding of gaining weight. This drawback is, however, an important aspect of this study. If the devices turn out to give patients more negative than positive effects, this will result in valuable information.

It can be stated that during the first years after the operation weight loss is majorly depending on the procedure itself and other factors, such as patients’ adherence or physical activity, only start to play an increasingly substantial role in weight sustain and regain after the first year. Therefore, it might be interesting to extend to study span to the full follow-up period of five years after the initial two-year study span.

### Trial status

The trial is ongoing and currently recruiting. Recruitment of participants started in February 2017.

## Additional files

Additional file 1: SPIRIT 2013 Checklist: Recommended items to address in a clinical trial protocol and related documents. (DOC 120 kb)

Additional file 2: Informed consent form (Dutch). (PDF 435 kb)

## Abbreviations

BMI: Body mass index
SAE: Severe adverse event
