# Supplementary material for: Assessing the value of eHealth for bariatric surgery (BePatient trial): study protocol for a randomized controlled trial
Source: Trials. 2018 Nov 14;19:625. doi: 10.1186/s13063-018-3020-x (PMC6237032; doi:10.1186/s13063-018-3020-x)
Supplement: Supplementary file 2 — Informed consent form (Dutch). (PDF 435 kb) [file 13063_2018_3020_MOESM2_ESM.pdf]

BEPATIENT -studie

De waarde van eHealth voor bariatrische chirurgie vaststellen

Ik heb de informatiebrief voor de proefpersoon gelezen. Ik kon aanvullende vragen stellen. Mijn vragen zijn genoeg beantwoord. Ik had genoeg tijd om te beslissen of ik meedoe.

Ik weet dat meedoen helemaal vrijwillig is. Ik weet dat ik op ieder moment kan beslissen om toch niet mee te doen. Daarvoor hoef ik geen reden te geven. Ik weet dat de onderzoeker en het behandelteam van het Obesitascentrum mijn gegevens kunnen zien.

Ik geef toestemming om mijn gegevens nog [15] jaar na dit onderzoek te bewaren.

Ik geef ☐ wel ☐ geen toestemming om mij na dit onderzoek opnieuw te benaderen voor een vervolgonderzoek

Naam proefpersoon	
Datum	
Handtekening	

---------------------------------------BEHANDELAAR---

Ik verklaar hierbij dat ik deze proefpersoon volledig heb geïnformeerd over het genoemde onderzoek. Als er tijdens het onderzoek informatie bekend wordt die de toestemming van de proefpersoon zou kunnen beïnvloeden, dan breng ik hem/haar daarvan tijdig op de hoogte.

Naam behandelaar	
Datum	
Handtekening	
